# Improving access to medicines by popularising generics: a study of ‘India’s People’s Medicine’ scheme in two districts of Maharashtra

**DOI:** 10.1186/s12913-022-08022-1

**Published:** 2022-05-13

**Authors:** Sonam Lavtepatil, Soumitra Ghosh

**Affiliations:** 1 School of Health Systems Studies, Tata Institute of Social Sciences, Mumbai, India; 2 Centre for Health Policy, Planning and Management, School of Health Systems Studies, Tata Institute of Social Sciences, Mumbai, India

**Keywords:** Access, Essential medicines, Essential medicine list, Affordability of generic medicine, Perception about generics, Pradhan Mantri Bhartiya Jan Ausadhi Pariyojana, India

## Abstract

**Background:**

In spite of being the ‘pharmacy of the world’, access to essential medicines for a large majority of Indians is constrained by both physical and financial reasons. According to an estimate, medicines account for 69% of household out-of-pocket spending on health care. To make quality generic medicine affordable, India’s People’s Medicine Scheme (*Jan Aushadhi*) was launched in 2008 and then revamped and rebranded as *Pradhan Mantri Bhartiya Jan Ausadhi Pariyojana* (PMBJP) in 2015. The current study focuses on the availability, affordability and acceptability aspects of PMBJP essential medicines.

**Methods:**

We have used a mixed-methods approach, with the survey-based quantitative component supplemented by a qualitative component consisting of in-depth interviews (IDIs). The survey was conducted in 11 PMBJP pharmacies in Mumbai and Palghar. Data were gathered on the availability, stock-outs, price and affordability of 35 essential medicines and 2 consumables.

**Results:**

Apart from the limited coverage of essential medicines and the significant presence of Fixed dose combinations (FDCs) in the PMBJP medicine list, the availability of surveyed essential drugs was also found to be low (47%) in PMBJP outlets. Across Mumbai and Palghar districts, around 50% and 42% of medicines were found to be out of stock for the period of 3–6 months respectively. The cost of generic medicines of PMBJP outlets for treating various conditions range from 0.01 days’ wages to 0.47 days’ wages for the lowest paid unskilled worker in Maharashtra.

**Conclusions:**

The study findings show that PMBJP’s unbranded generics offer great opportunities for substantial cost savings. But, in order to fully realise the potential of this scheme, some policy actions are urgently required. First, the PMBJP drug list must include all essential drugs that feature in NLEM. Second, BPPI should procure only those drugs that pass the bioequivalence test. Third, compulsory de-branding of generics should be done in a phased manner. Fourth, PMBJP’s medicine procurement and distribution policies must be reviewed to address the supply chain issues. Moreover, there is a need for major pharmaceutical policy reforms to promote generic medicines in a big way. Regulations to support mandatory generic prescribing and generic substitution by pharmacists are needed.

**Supplementary Information:**

The online version contains supplementary material available at 10.1186/s12913-022-08022-1.

## Background

Lack of access to essential medicines is a major health policy concern globally, even more so in the low-and middle-income countries. According to the World Health Organisation, a whopping 2 billion people worldwide are not having access to essential medicines [1]. In other words, drugs that can extend lives by stopping deadly diseases like cancer or AIDS, provide relief from excruciating pain caused by preventable and curable diseases are not available and accessible to more than a quarter of the world population. In India, despite the presence of a thriving generic pharmaceutical industry, a principal barrier to access continues to be the cost of medicines. According to WHO Figs. (2004), 65% Indians or nearly 650 million lacked access to essential medicines and medicine constitutes 63% of household’s total out-of-pocket (OOP) health payments, thereby impoverishing millions of people every year [2, 3].

The issue of access to medicine is a primary cause of the skewed healthcare utilisation pattern in India. According to National Sample Survey Organisation, public facilities accounted for only 30% of the overall healthcare services in the year 2017–18 [4]. This means that majority of the people had to obtain healthcare including medicines from private providers. Moreover, even those who access healthcare from public facilities end up purchasing drugs from the market as the prescribed medicines are often not available in pharmacies of public hospitals. According to an estimate, access to essential medicines is below 35% in India [5].

### Medicine pricing in India

In India, prices of most drugs are fixed by the firms that manufacture or import the drugs into the country. Stated differently, prices of drugs are largely determined by market conditions and market forces. Only those drugs which feature in the National List of Essential Medicines (NLEM) come under price control [6]. As of July 2021, price ceiling has been imposed on 355 bulk drugs and their formulations from the NLEM 2011 as per the provisions of Drugs (Price Control) Order (DPCO),^1^ 2013 [7]. According to an estimate, about 14% of the Rupees 1.36 trillion (US $18.4 billion^2^) domestic pharmaceutical market are under price control (25% by volume) [8]. National Pharmaceutical Pricing Authority (NPPA) sets the ceiling prices^3^ for scheduled or essential medicines following a market**-**based pricing (MBP) methodology. That apart, for all non-scheduled drugs, the NPPA merely monitors their prices and an annual increase in the maximum retail price of up to 10% is allowed for such medicines.

A large number of observers branded the NLEM 2011 as irrational and have also been critical of the adoption of MBP approach by DPCO, 2013, abandoning the earlier cost-based pricing mechanism, for fixing the prices of essential drugs, arguing, that the impact of price control would be to make drugs available at reasonable prices but would at best partially address the issue of affordability and access to medicine as MBP has no relation with the cost of medicines [9–11]. It was argued that, in case of market based ceiling price, medicines would be way too expensive than under the cost-based pricing approach followed by DPCO, 1995 [12]. That the prices of drugs fixed by NPPA remained high and majority of medicines including several essential and lifesaving drugs were kept out of DPCO posed a challenge in terms of making the drugs affordable and accessible to the people [12, 13].

Against this backdrop, in September 2015, the union government decided to expand its generic drug scheme called “*Pradhan Mantri Bhartiya Jan Aushadhi Pariyojna* (PMBJP)”, which envisaged making unbranded quality-assured generic medicines^4^ available at affordable prices to all people and especially the poor. Further, in order to generate demand for unbranded generic medicines, in 2017, the erstwhile Medical Council of India issued a circular to the medical fraternity to comply with its regulation for prescribing medicines by generic names [14]. Besides, since April 2017, bioequivalence studies have become an essential requirement for the manufacture of a generic medicine in India [14]. While the idea of popularising non-branded generic drugs is a robust policy response to improve access and reduce pharmaceutical spending, little is known about how far these initiatives have been effective in making generic drugs accessible at affordable prices.

### Evolution of PMBJP

The introduction of PMBJP is not so recent. PMBJP, originally called *Jan Aushadhi Scheme* (JAS), was initiated by the United Progressive Alliance (UPA) Government in 2008. JAS, as is argued, is an important government intervention in the pharmaceutical market which would make the supply side effective particularly for consumers who are relatively responsive to price changes in making their purchasing decisions of medicines [15]. The Bureau of Pharma Sector Undertakings (BPSU) working under the Department of Pharmaceuticals, Government of India, was entrusted with the responsibility of implementing JAS, i.e., to coordinate procurement, supply and marketing of generic drugs through JAS outlets. The scheme envisaged to sell generic medicines at affordable prices through exclusive outlets namely JAS stores across the country, starting from the district to sub-divisional headquarters and to towns and villages. However, JAS never really took off; there were only 99 JAS outlets across India which were selling 131 medicines till 2014. This was reportedly due to a number of reasons such as excessive dependence on state government for the implementation of the scheme, lacunas in supply chain operations, physicians’ reluctance to prescribe generic medicines, distribution of free medicines through state-sponsored schemes and poor level of awareness among the people [13–15]. According to a study, the availability of medicines in JAS was abysmally low (33%) [13, 14].

After a change in the political leadership at the centre, in 2015, JAS was renamed. Aside from changing the scheme’s name, BPPI tweaked the programme a bit with a ‘new strategic action plan’ for the effective implementation of PMBJP, which included increased financial support to the store owners, greater incentives for opening outlets in disadvantaged locations, upward revision of trade margins to retailers and distributors and an expanded product basket.

This study reported the availability, stock-outs and affordability of a basket of essential medicines (unbranded) and consumables in selected PMBJP stores in the districts of Palghar and Mumbai in Maharashtra. Besides affordability, we did cost comparison of unbranded and branded generic equivalents of some commonly used medicines. Previous studies suggest that doctors’ negative perceptions regarding generics was a major constraint faced by JAS leading to its poor success [15,]. PMBJP’s stated objective is to gain the confidence of the medical community and consumers in unbranded generics by generating awareness through education and publicity. In this study, we have assessed the acceptability of PMBJP’s unbranded generics It is worth noting that the Indian pharmaceutical market is flooded with irrational or non-essential drugs. For example, majority of fixed dose combinations (FDC) that are marketed in India are therapeutically non-beneficial and unsafe for use [16, 17]. Notwithstanding, such FDCs account for more than 50% of the pharmaceutical formulations in India [17]. We, therefore, critically looked at the selection criteria for medicines included in the PMBJP list. In addition, views of healthcare professionals regarding unbranded generic medicine were studied.

### Methodology

We have used a mixed-methods approach, with the survey-based quantitative component supplemented by a qualitative component consisting of in-depth interviews (IDIs). The survey was undertaken to find out the extent of availability and stock-out of medicines at PMBJP outlets. In-depth interviews were intended to capture the perspectives of physicians and pharmacists on generic medicine in general and PMBJP scheme in particular.

#### Setting of the study and sampling

The survey was conducted in two districts of Maharashtra, namely Mumbai metropolitan region and Palghar. In terms of per capita income, Maharashtra is one of the richer states in India and Mumbai is its capital city. With a population of more than 20 million, the city is one of the most populous urban centres in the world. It has the distinction of being home to the largest slum population in any city in the world, displaying a high level of income inequality. On the other hand, Palghar is an economically backward district with a population of nearly 3 million and is primarily inhabited by the tribal people.

**Fig. 1:**
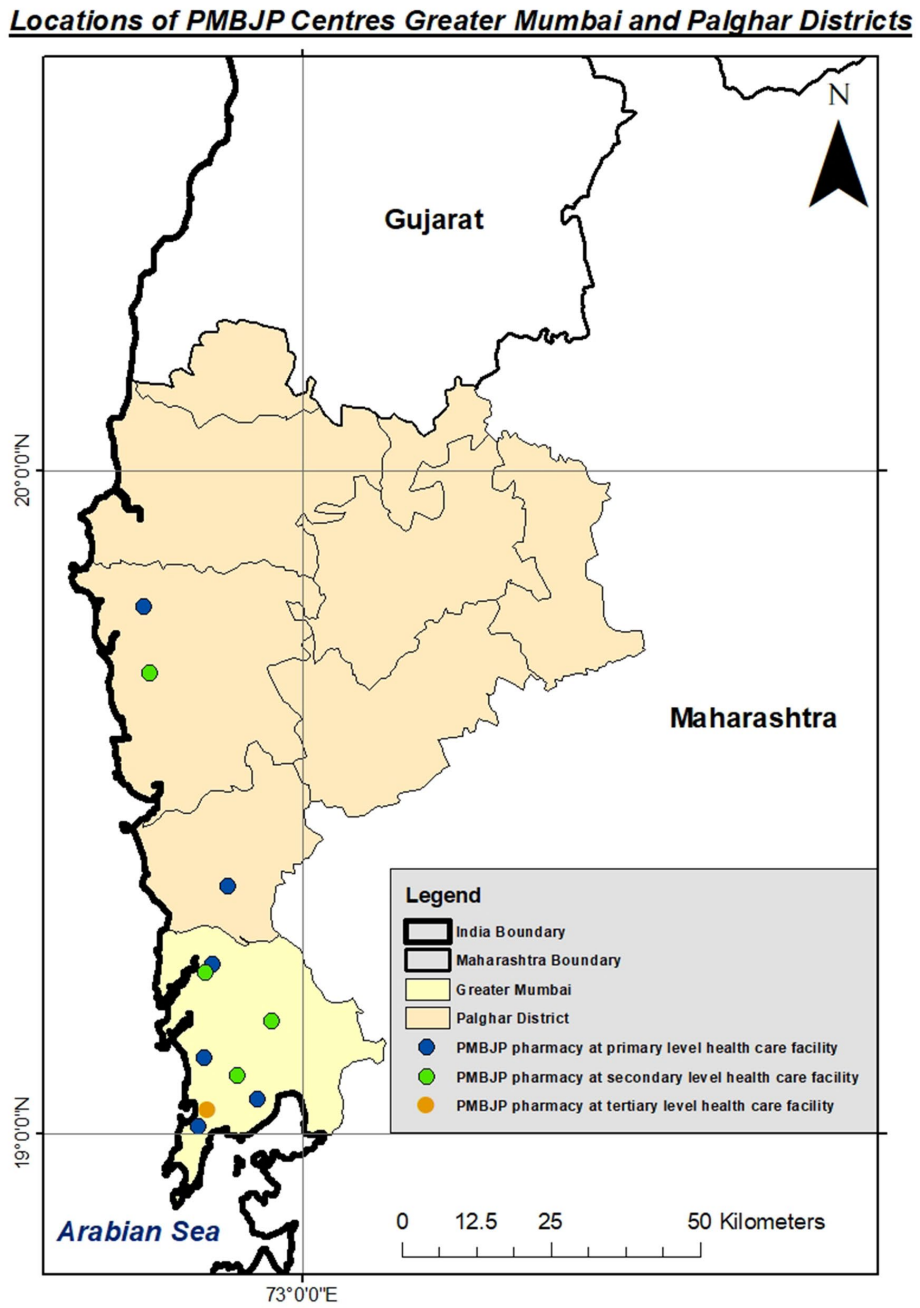
Map showing locations of PMBJP pharmacies across Mumbai metropolitan and Palghar region. □: PMBJP pharmacy at tertiary level of health care, □: PMBJP pharmacy at secondary level of health care, □: PMBJP pharmacies at primary level of health care

A 3-tiered public healthcare delivery system is catering to the needs of the population of these two districts. At the bottom of the health services pyramid, there are primary health centres (PHC), sub-centres (SC) and health posts (HP), which focus on primary care. The mid-level of the pyramid, which is the secondary care level, includes rural hospitals and municipal general hospitals, and at the top level, tertiary care institutions like medical colleges are there. According to the PBBJP portal, 29 PMBJP pharmacies are currently functional across these two districts. To make the sample representative and consistent with the distribution of PMBJP outlets in Mumbai and Palghar, 11 pharmacies were selected for the study covering all levels of health care delivery system. The PMBJP pharmacies were purposively selected based on their proximity to public health facilities such as medical college (Tertiary level), Municipal General Hospital (Secondary Level) and PHCs and HPs (Primary level). PMBJP pharmacy’s proximity to the public health facilities was assessed using google maps (Fig. 1). The details of the sampled PMBJP pharmacies are provided in Table 1. Our sample represented all PMBJP pharmacies in each of the study districts. All the selected PMBJP outlets agreed to participate in the study; and a written consent form was signed before data collection.

**Table 1:** Sampling of PMBJP pharmacy across different levels of health facility

Level of health care	Types of health facility considered for the study	Universe of the study (Total number of PMBJP kendras at each level of health system)	No of health Facility considered for the study	No. of PMBJP Kendra selected for the study according to the proximity of health facility
**Tertiary level of health care**	Medical College and Government Hospital (Sion Hospital)	01	01	01
**Secondary level of health care**	Mumbai Municipal Corporation General Hospital/ Peripheral hospital (Ghatkopar, Borivali, Mulund)	11	03	03
	Rural Hospital (Palghar)	01	01	01
**Primary level of health care**	Health Post (Mankhurd, Dahisar, Andheri, Mahim)	12	04	04
	Primary Health Centre (Vasai, Nalasopara nirmal PHC)	04	02	02
	**Total**	**29**	**11**	**11**

### Selection of medicines for the survey

The selection of essential medicines, for our survey, was a two-step process. We first reviewed the WHO-HAI core list of medicines, medicines that are listed on India’s Essential Medicine List and drugs listed under various national health programmes [18, 19]. Following this, we picked the essential drugs from the PMBJP product basket, which were found on all the above-mentioned lists. Of the 35 surveyed medicines, 22 were from the first (for global burden of disease) and second (specific to Southeast Asia) core medicine lists recommended by WHO/HAI methodology and 13 were drawn from the NLEM, 2015. Information was obtained on the availability of essential medicines at PMBJP outlets across primary, secondary and tertiary levels of health care delivery system against the basket of 22, 28 and 35 medicines respectively selected according to therapeutic category. Among the medicines included in the survey, 22 are universal medicines, which are supposed to be available at all levels. Apart from the universal medicines, 6 more medicines were considered at the secondary level, while, in addition to the medicines at the primary and secondary levels, 7 other medicines were assessed for availability at the tertiary level. Along with medicines, availability of 02 consumables was checked. The list of medicines and consumables has been provided as an additional file (Additional file 1).

### Data collection

A structured tool was used to gather information on the availability of survey medicines and consumables on the day of survey, medicine stock-out status for the period of six months preceding the date of survey and the time span of stock-outs at PMBJP drug stores at different levels of health care system in two sample districts of Maharashtra. In order to collect data in an accurate and reliable manner, one of the researchers personally visited the selected PMBJP outlets to check the availability of generic medicines. The researcher cum field investigator is trained in both pharmacy and public health with previous experience of conducting primary data collection for health systems research. Data were extracted from stock registers kept in PMBJP pharmacies. She used the structured schedule to collect data on availability and stock-out of selected medicines and consumables. Those medicines not found in stock on the day of survey, the number of days of stock-outs in the last 6 months were recorded through manual checking of registers.

### Qualitative data

Interview guides were used to conduct in-depth interviews of physicians and pharmacists. We conducted a total of 16 in-depth interviews (IDIs) – 10 with pharmacists and 6 with physicians. Purposeful sampling was used for identifying and selecting the participants for IDIs. Physicians working in public or private sector and practicing in the periphery of PMBJP pharmacies were selected for the study. Only qualified allopathic doctors and the pharmacists registered under the state or central pharmacy council were considered for IDIs. Both quantitative and qualitative data were collected concurrently between January, 2019 and June, 2019.

Aside from collecting primary data, secondary data such as the price information of some PMBJP medicines and their leading brand name counterparts (in terms of market share) were gathered from the web portals of PMBJP and MedGuideIndia.com respectively. The latter is a project of Vinod Kumar Memorial Trust. The website provides information on a wide range of drugs available in the Indian pharmaceutical market including their prices. The website is regularly updated by the Trust with assistance from Ministry of Consumer Affairs, Government of India.

### Data analysis

SPSS version 21.0 and MS Excel were used to analyse the quantitative data to measure theavailability, and affordability of essential medicines of PMBJP. Availability was defined as the proportion of PMBJP pharmacies in which the medicines were available at the time of the survey. We calculated the availability of medicines according to a specific therapeutic category. Hence, the medicines were grouped into therapeutic categories. The formula used for calculating availability is as follows: (n/N) X100, where n is the number of medicines available within a specific therapeutic category in a PMBJP pharmacy on the survey date and N referred to total number of drugs listed in that category as per the survey medicine list. For a PMBJP pharmacy at specific level of care (e.g. primary health care level in Palghar), overall availability of medicine from specific therapeutic category was calculated using the following formula:

$$\frac{\sum \left({n}_{i}\right)\mathrm{X}100}{PXN}$$

Here, *ni* was the number of drugs available within a particular therapeutic category at specific PMBJP pharmacy and P was the number of PMBJP pharmacies at specific level of care (here, 02 PMBJP pharmacies at PHC level from Palghar were visited and surveyed) and N was the total number of drugs listed in particular therapeutic category as per the survey medicine list. Hence, N X P gave the total number of drugs in that therapeutic category which were surveyed at all PMBJP pharmacies at PHC level in Palghar. For instance, if availability of 7 (N) antimicrobials medicines in 02 PMBJP pharmacies (P) at PHC level in Palghar was evaluated, then overall 14 units (PXN) would be examined. Against a group of these units, if 03 antimicrobials medicines were available in a PMBJP store and 04 antimicrobials medicines were available in another store, then the overall availability of anti-microbials in PMBJP pharmacies at primary level facilities of Palghar was 50% [((3X1) + (4X1)) X100/14].

Total availability of drugs from specific therapeutic category in all PMBJP pharmacies across three levels of health services system in a study area was calculated using the following formula:

$$\frac{\sum \left(\mathrm{ni }\right)*100}{\sum \mathrm{Pi}*Ni}$$

i was in the range of 1–3, where 1,2,3 represented three different health care facility levels such as primary (Health Post in urban area /PHC in rural area), secondary (District Hospital/Peripheral Hospital) and tertiary (Medical college & hospital). Here, ni was the number of drugs from a specific therapeutic category available in a PMBJP pharmacy at a particular health care level and Pi was the number of facilities in that particular level of care and Ni was the total number of drugs listed in particular therapeutic category as per the survey medicine list. Similarly, stock-out of medicines was calculated for the reference period of six months.

### Costs and affordability

To demonstrate the opportunities for potential cost savings associated with the use of unbranded generics, we calculated the costs in treating patients for certain diseases with the PMBJP drugs vis-à-vis their largest selling branded-generic counterparts (popular brands). We chose the latter for comparison as it helps to gauge the potential savings that can be earned by the consumers from substituting branded drugs by the unbranded drugs of PMBJP. As far as the measurement of affordability of essential medicines is concerned, we followed the WHO/HAI methodology. Affordability was estimated by comparing the total cost of a PMBJP medicine for a standard course of treatment for common acute and chronic conditions to the daily wage of the lowest paid unskilled worker, which was supposed to be Rs. 355 in Maharashtra in 2019, as per the state government’s notification on minimum wages [20].

### Acceptability of PMBJP medicine

The qualitative data was collected to understand the perception of the doctors and pharmacists on generic medicine as well as their views on PMBJP scheme. Following informed consent, the interviews were audiotaped while the first author also took notes. Recordings were translated from the local languages such as Marathi and Hindi in English, and then transcribed verbatim. We used thematic analysis [21], which warranted reading and rereading the transcripts in order to generate the potential themes. Coding of the materials and categorization were done to review and revise the themes. The profiles of the physicians and pharmacists are provided as additional files (Additional files 2 and 3).

## Results

### Selection of rational, affordable essential medicines and quality assurance criteria for PMBJP medicine list

#### Coverage of essential medicines in PMBJP

**Fig. 2:**
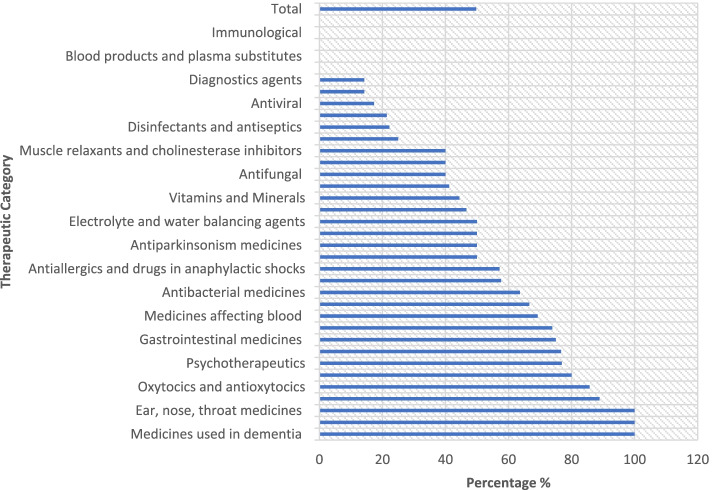
Coverage of essential medicines under PMBJP

We first examined the selected medicine list of PMBJP to assess the extent of coverage of essential medicines. NLEM, 2015 has 376 essential drugs whereas the PMBJP list contains 214 essential medicines. In other words, the overall coverage of essential drugs in PMBJP list is just 57%, when compared to the total number of essential medicines included in NLEM. Moreover, there is significant variation across therapeutic categories (Fig. 2).

#### Presence of non-essential FDCs and unsafe drugs under PMBJP product list

WHO model list of essential medicines has total 37 FDCs, while NLEM, 2015 consists of 24 FDCs. As far as the NLEM list is concerned, majority of these FDCs are aimed at improving treatment adherence and preventing drug resistance in diseases of public health concerns such as malaria, TB and HIV-AIDS. However, as shown in Table 2, PMBJP medicine list has as many as 130 FDCs in different therapeutic categories. The maximum number of fixed dose formulations listed in PMBJP is for vitamins and micronutrient deficiency followed by gastrointestinal complaints and cardiovascular disorders.

**Table 2:** Comparison between PMBJP medicine list and NLEM, 2015 in terms of number of FDC drugs by therapeutic group

Therapeutic Category	Number of FDCs in PMBJPY List	Number of FDCs in NLEM, 2015
Analgesics, antipyretics and NSAIDs	15	–––
Anaesthetic	–––	02
Anthelminthic	01	–––
Antiallergics	08	–––
Antibacterial	13	03
Antifungal	01	–––
Antiprotozoal	01	02
Antiviral (Anti-HIV)	–––	09
Antiseptic	02	–––
Anti-tuberculosis medicines	01	–––
Anti-Parkinson’s	–––	01
Hormones, other endocrine medicines & contraceptives	07 (Antidiabetic)	02
Cardiovascular medicines	15	–––
Dermatological	08	–––
Ear, nose, throat medicines	02	–––
Gastrointestinal medicines	20	–––
Medicines acting on the respiratory tract	04	01
Cholinesterase inhibiting agents and Muscle relaxants	02	01
Oxytocic and Antioxytocics	02	–––
Psychotherapeutics	06	–––
Immunological	–––	02
Electrolyte and water level correcting agents	–––	01
Vitamins and minerals	22	01
**Total**	**130**	**24**
**Unsafe / Banned drugs**	**01**	–––

### Availability of essential generic medicines at pmbjp pharmacies/ kendras across mumbai and palghar region

Table 3 shows the extent of availability of medicine at PMBJP pharmacies across all levels of care in Mumbai and Palghar**.** Overall, the mean availability of medicines across study districts was found to be 52%, though the mean availability of medicine was slightly higher in Palghar (54%) than in Mumbai (51%). The findings suggest that availability of medicines vary significantly, ranging from 8 to 72% in Palghar and from 0 to 83% in Mumbai. Table 4 displays the extent of availability of medicines in Palghar district. The mean drug availability at PMBJP stores located in the vicinity of PHCs and district/rural hospitals was 51% and 61% respectively. While more than 70% of PMBJP pharmacies across all levels of care in Palghar region had palliative care medicines, anti-epileptics, analgesics and antacids, the availability of anti-cancer, anti-asthmatic and electrolyte balancing drugs were found to be very poor (less than 30%).

**Table 3:** Availability of medicines from various therapeutic categories at PMBJP stores by facility level in Palghar and Mumbai, 2019 (in percent)

Drug Category	Palghar			Mumbai			
	**Primary**	**Secondary**	**Total**	**Primary**	**Secondary**	**Tertiary**	**Total**
	**(*P* = 2)**	**(*P* = 1)**	**(*P* = 3)**	**(*P* = 4)**	**(*P* = 3)**	**(*P* = 1)**	**(*P* = 8)**
**Antimicrobial**	45.23	50	**46.7**	47.6	52.3	54.7	**50.8**
**Cardiovascular**	62.49	79	**67.5**	56.5	79.5	77.7	**69**
**Antidiabetics**	44.44	61	**49.9**	52.7	53.6	49.9	**52**
**Drugs used in Palliative Care**	66.66	83	**72**	54	44.4	50	**50**
**Anticancer**	0	33	**11**	0	27.7	49.9	**17**
**Antipsychotics**	25	100	**50**	12.5	50	50	**31**
**Antiepileptics**	95.83	50	**80.2**	70.8	16.66	0	**48**
**Antiasthamatics**	37.5	16.6	**24.8**	43.75	19.4	0	**29**
**Analgesics**	91.66	100	**94.4**	85.4	83.3	50	**78**
**Antacids**	79.16	92	**83**	79	83.3	83.3	**81**
**Medicines used for electrolyte imbalance**	0	25	**8.3**	12.5	22.2	33.3	**18**
**Vitamins**	62.75	83	**69.3**	81.2	63.8	66.6	**73**
**Consumables**	75	41	**63.7**	62.5	49.9	41.6	**60**
**Total**	**51.12**	**61.33**	**54.5**	**48.75**	**52.55**	**50.89**	**51**

^*^P = Number of sampled PMBJP pharmacies at different health care level

**Table 4:** Extent of availability of surveyed medicines at Palghar region

Low availability (< 35%)	Moderate availability (40–65%)	High availability (more than 65%)
Anticancer (11%)	Antimicrobial (44%)	Cardiovascular (68%)
Antiasthamatics (25%)	Antidiabetics (49%)	Palliative care medicines (72%)
Medicines used for electrolyte imbalance (8%)	Antipsychotic (50%)	Antiepileptics (80%)
––––––	Consumables (64%)	Analgesic (94%)
–––––-	–––––-	Antacids (83%)
–––––-	–––––-	Vitamins (69%)

Table 5 shows the extent of availability of surveyed medicines in Mumbai. As mentioned earlier, overall, mean drug availability was found to be around 51% with highest at PMBJP pharmacies at secondary level of care i.e., at peripheral hospitals (52.5%), followed by tertiary level i.e. medical college (50.8%) and primary level i.e. health post (48.7%). More than 70% of PMBJP pharmacies across all levels of care had the medicines in categories of cardiovascular, analgesics, antacids and vitamins. On the other hand, the availability of antiasthmatic and antipsychotic medicines was very low-only a third of the PMBJP pharmacies had them. Anticancer and electrolyte balancer were not available in more than four-fifth of PMBJP stores, while half of the pharmacies had antimicrobial, antidiabetics, palliative care medicines, anti-epileptics.

**Table 5:** Extent of availability of medicines at Mumbai

Low availability (< 35%)	Moderate availability (40–65%)	Highest availability (more than 65%)
Anticancer (17%)	Antimicrobial (51%)	Cardiovascular (69%)
Antipsychotic (31%)	Antidiabetics (52%)	Analgesic (78%)
Antiasthmatic (29%)	Palliative care medicines (50%)	Antacid (81%)
Medicines used for electrolyte imbalance (18%)	Antiepileptics (48%)	Vitamins (73%)
––––––	Consumables (60%)	–––––

#### Stock-out duration of medicines in PMBJP pharmacies of Palghar

**Fig. 3:**
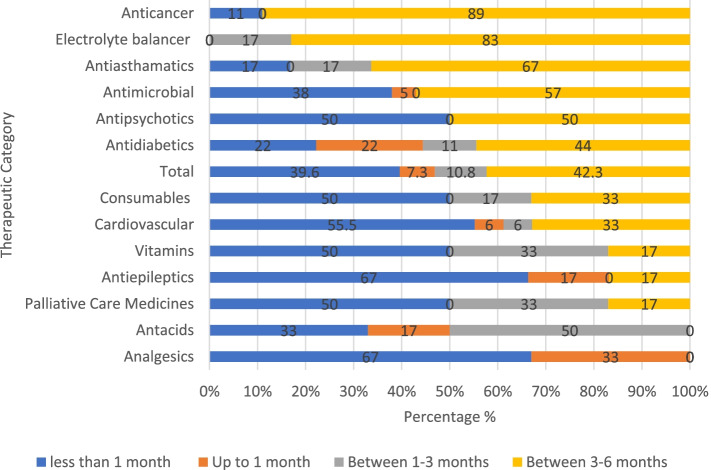
Duration of stock-outs for medicines at PMBJP stores, Palghar

Among the medicines unavailable in PMBJP stores at the time of the survey, around 42% were out of stock for the period of 3–6 months, while 11% and 7% of drugs were found to be out of stock for 1–3 months and up to 1 month respectively (Fig. 3). In Palghar, about 86% of anticancer and electrolyte balancing medicines, which were unavailable at the time of survey, were out of stock for 3–6 months. Among the antidiabetics, cardiovascular and consumables not available on the survey day, 44% and 33% respectively were out of stock for the period of 3–6 months. Nearly 30% of palliative care medicines and vitamins, about 17% of antiasthmatics and consumables were out of stock for a period of 1–3 months.

#### Duration of stock-outs of medicines in PMBJP pharmacies of Mumbai, 2019

**Fig. 4:**
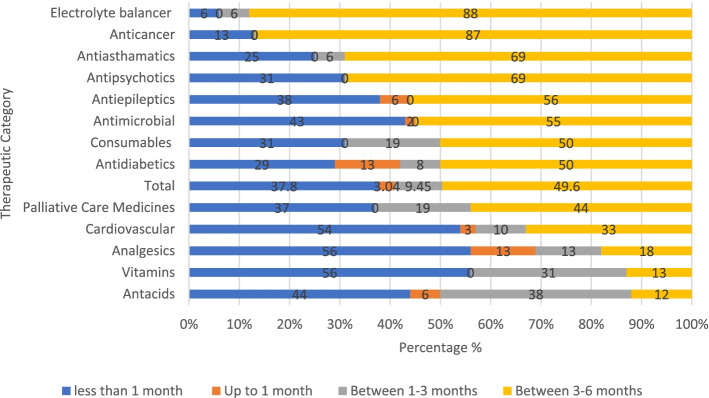
Duration of stock-outs of medicines in PMBJP Stores, Mumbai

In Mumbai region, among the surveyed medicines which were not available at the time of survey, around 50% were out of stock for the period of 3–6 months while about 38% of medicines were out of stock for the period of less than 1 month (Fig. 4). Around 50% of antimicrobials, antidiabetics, antiepileptics and consumables were out of stock for the period of 3–6 months. Almost 70% of antipsychotics and antiasthmatics, more than 85% of anticancer and electrolyte balancing medicines were found to be out of stock for 3–6 months.

### Scope for costsavings on medicine and affordability of PMBJP medicines

Table 6 shows the cost of standard treatments, as recommended by WHO, with surveyed medicines at PMBJP price and at branded generic price for a number of health conditions [22, 23]. We find that for all selected disease conditions, the drug cost of the thirty-day treatment reduce by 6–1129% if PMBJP’s unbranded medicines are used instead of their largest selling branded-generic counterparts. For the treatment of Type 2 diabetes, the monthly expenditure on the PMBJP medicine (Glimepiride) was estimated to be almost 50% lower compared to its equivalent branded-generic counterpart. We further observed that the PMBJP drug (enalapril) used for the treatment of hypertension costs 725% lower per month than that of the reference branded-generic drug.

**Table 6:** Cost of standard treatments for selected chronic and acute illnesses using PMBJP drugs and largest selling branded generic medicines

Sr. No	Condition	Medicine	Strength	Dosage Form	Standard Treatment	Cost of unit x dosage frequency (in Rs.)		
						Cost as per MRP of popular brand	Cost of PMBJP drugs	Cost Difference [7-8] (%)
(1)	(2)	(3)	(4)	(5)	(6)	(7)	(8)	(9)
1	Asthma	Salbutamol	o.1 mg/ dose	Inhaler	1 inhaler of 200 doses	105	37	68 (184)
2	Diabetes	Glimepiride	1 mg	Cap/ Tab	1 cap/tab x 2 /day × 30 days = 60	73	24	49 (204)
3	Hypertension	Atenolol	50 mg	Cap/ Tab	1 cap/tab × 30 days = 30	88.92	84	5 (6)
4	Hypertension	Enalapril	5 mg	Cap/ Tab	1 cap/tab x 2 /day × 30 days = 60	198.48	24	174 (725)
5	Hypercholesteremia	Atorvastatin	10 mg	Cap/ Tab	2 cap/tab x 2 /day × 30 days = 120	1031.92	84	948 (1129)
6	Anticoagulant	Clopidogrel	75 mg	Cap/ Tab	1 cap/tab x 2 /day × 30 days = 60	823.14	90	733 (814)
7	Adult Respiratory tract infection	Ciprofloxacin IP	500 mg	Cap/Tab	1 cap/tab x 3 /day × 7 days = 21	138.60	36	103 (286)
8	Adult Respiratory tract infection	Ceftriaxone	1 g/vial	Injection	1 injection	160	24	136 (567)
9	Adult Respiratory tract infection	Amoxicillin	500 mg	Cap/Tab	1 cap/tab x 3 /day × 7 days = 21	128	61	67 (110)
10	Anxiety	Diazepam	5 mg	Cap/Tab	1 cap/tab x 7 days = 7	19.42	4	15.4 (385)
11	Arthritis	Diclofenac	50 mg	Cap/Tab	1 cap/tab x 2 /day × 30 days = 60	166.26	18	148.3 (824)
12	Depression	Amitriptyline	25 mg	Cap/Tab	1 cap/tab x 3 /day × 30 days = 90	179	169	10 (6)
13	Ulcer	Omeprazole	20 mg	Cap/Tab	1 cap/tab × 30 days = 30	240	21	219 (1043)

Maximum retail price (MRP) is the price to patient and is printed on the package

**Table 7:** Affordability of PMBJP medicines for standard treatments for particular health conditions in terms of daily wages

Sr. No	Condition	Treatment	Affordability at MRP/ day wage	Affordability at PMBJP/ day wage
1	**Anxiety**	**Diazepam 5 mg Cap/Tab**	**0.05**	**0.011**
2	**Diabetes**	**Glimepride 1 mg Cap/Tab**	**0.2**	**0.067**
3	**Hypertension**	**Atenolol 50 mg Cap/ Tab**	**0.25**	**0.236**
4	**Asthma**	**Salbutamol 0.1 mg/ dose inhaler**	**0.295**	**0.1**
5	**Adult Respiratory tract infection**	**Amoxicillin 500 mg Cap/ Tab**	**0.36**	**0.17**
6	**Adult Respiratory tract infection**	**Ciprofloxacin IP 500 mg Cap/ Tab**	**0.39**	**0.101**
7	**Adult Respiratory tract infection**	**Ceftriaxone 1 g/ vial injection**	**0.45**	**0.06**
8	**Depression**	**Amitryptyline 25 mg Cap/ Tab**	**0.46**	**0.05**
9	**Arthritis**	**Diclofenac 50 mg Cap/ Tab**	**0.5**	**0.476**
10	**Hypertension**	**Enalapril 5 mg Cap/ Tab**	**0.55**	**0.067**
11	**Ulcer**	**Omeprazole 20 mg Cap/ Tab**	**0.67**	**0.05**
12	**Anticoagulant**	**Clopidogrel 75 mg Cap/ Tab**	**2.31**	**0.253**
13	**Hypercholestermia**	**Atorvastatin 10 mg Cap/ Tab**	**2.9**	**0.236**

**Fig. 5:**
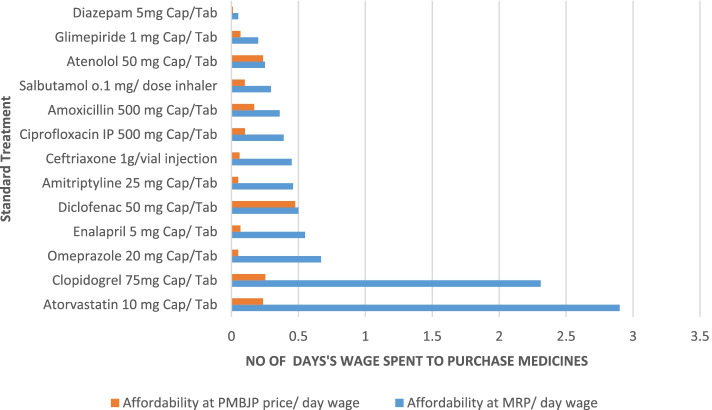
Affordability to medicines at MRP and PMBJP prices

Affordability was calculated on the basis of number of days of wages that an unskilled worker requires to expend for a standard course of treatment for common acute and chronic conditions [22, 23]. As shown in Table 7 and Fig. 5, the cost of the treatment with PMBJP medicines was between 0.01 days’ wages and 0.48 days’ wages, suggesting that unbranded generic medicines at PMBJP pharmacies were relatively affordable than their branded counterparts. At 0.01 and 0.5 day’s wage, diazepam for treating anxiety and diclofenac for treating arthritis respectively were the cheapest generic medicines at PMBJP stores. The maximum price differential was observed for atorvastatin and clopidogrel, used for treating hypocholesteraemia and clopidogrel respectively, indicating that the unbranded medicines can be fairly affordable.

#### Findings from qualitative data

##### Perceptions regarding generic medicines among physicians

Assessing the availability and affordability of generic medicine at PMBJP outlets is important, but perhaps it is even more important to know the physicians’ attitude towards generic drugs and PMBJP scheme, as they play a key role in prescribing generic medicines. Most of the physicians argued that generic and branded medicines have the same active substance(s).

*“Actually, both are same. Nothing is different in generic and branded except cost. The important part of medicine i.e. molecule/drug is same in both the cases.” (PuP1)*

Nevertheless, they claimed that generic medicines are not as effective as branded medicines. They also believed poor bioavailability is the reason why the price of generic medicines is less than branded medicines.

*“Bioavailability of generic medicines is low as compared to branded medicines. Pharmaceutical companies market their brands by branding their bioavailability results. Poor bioavailability makes generic medicine less effective. Hence cost of generic medicines is lower than their branded counterparts.” (PuP3).*

##### Generic drug prescribing experience

Clinicians working in the public sector said that they prescribe 99% medicines in generic form as the government supplies only generic medicines to public health facilities. Further, the healthcare professionals stated that generics do not yield good results while treating for acute conditions like bacterial infections, but they expressed confidence in generic medicines for chronic illnesses*.*

* “Generics are good, but some products are not up to the mark. Some brands of antibiotics are found to be more effective as compared to their generic counterparts. For example, augmentin brand gives effective and quick results as compared to its simple generic amoxicillin clavulanate version. But generic medicines for diabetes and hypertension are very good and cost effective for the patients for chronic illnesses.” (PuP3).*

Some physicians reported that they face difficulties in prescribing generics as patients have negative attitudes towards cheaper medicines.

*“We are ready to prescribe low cost generics, but it becomes difficult for me to convince my patients to take such a low-cost generic medicine. Most of the time patients want relief from pain as quick as possible and thus they are ready to pay even high for medicines. Hence, they question about quality and their effectiveness.” (PrP2).*

In fact, the physicians have also cited other reasons for not prescribing generic medicines. The pharmacists, they claimed, will sell noneffective, or highly overpriced drugs once the authority to decide the drug for a given indication is bestowed upon the pharmacists through the policy of compulsory active ingredient prescribing.

*“We are ready to prescribe generic only, but there is no mechanism to ensure that private pharmacy shop chemists would dispense right and cost-effective generic medicines. Because there are hundreds of generics with different prices are available in the market, chemists can substitute for prescribed medicine as per their wish and earn high profit margins. There is no proper communication channel between doctors, patients and pharmacists. The loop of three is incomplete.” (PrP3).*

Furthermore, the physicians expressed the need for developing a regulatory framework to contain the proliferation of brands by the firms operating in the Indian pharmaceutical market, which, according to them, pose a barrier in prescribing generic medicines.

*“We are prescribing generic drugs, but through their brand names. If government wants us to prescribe drug through their molecule names, why do they allow so many brand names and why are there such wide price variations?” (PuP2).*

##### Views on PMBJP scheme

The physicians, especially those from the rural Palghar district wondered why the generic drug stores are not opened in rural areas where it is needed more. Besides that, the public physicians stressed on the need for establishing the PMBJP outlets within the hospital premises, while private physicians asked for dissemination of information regarding the drugs listed under the PMBJP scheme.

* “When we write any generic medicines and if it is not available in hospital pharmacy, patients have to go to other pharmacies to buy it. If these Jan Aushadhi stores are available in Government hospital premises, patient can get those medicines easily.”(PuP2).*

Further, the private physicians underlined the need for developing strong regulatory mechanisms to ensure that generic drugs meet high standards of quality.

*“Unless doctors are not getting convinced over quality, I don’t think they are going to routinely write out prescriptions with generic drugs.” (PrP3)*

##### Pharmacists’ views on PMBJP

In-depth interviews with pharmacists revealed about the non-supportive attitude of physicians practicing in the periphery of PMBJP stores. Pharmacists expressed their views as follows,

* “Doctors practicing here are not responding well to generic medicines. Doctors did not seem to be very much confident about the effectiveness of generic medicines. Doctors ask us to ensure efficacy of generic medicines by claiming it in writing.” (P8).*

*“We did Jan Aushadhi scheme awareness campaign twice in the area with the help of Regional Marketing Head of Mumbai region for Jan Aushadhi scheme, we visited doctors practicing in the periphery of our Jan Aushadhi stores. But we didn’t receive cooperation from doctors.” (P10).*

Further, the functioning of PMBJP scheme is often disrupted by supply chain shortage from causes such as procurement or/and distribution issues.

*“Now what happens is most of the time we don’t get adequate supply of these Jan Aushadhi products in definite time. Because medicines are coming all the way from Delhi, which takes time. In this case, we have to rely on our own network. Hence, government can increase number of wholesalers, distributors in the scheme to improve availability of medicines at the stores.” (P9).*

One of the major consequences of supply chain disruptions is reputation impact.

*“Patients are attracted towards this scheme due to mass media campaign on PMBJP scheme, but most of the time there is lack of supply of medicines.” (P5)*

Some reported that quality issues are hindering the uptake of unbranded generics, and asked for strong regulatory framework for quality control.

*“Some generic medicines are not of good quality. In many cases the generic drugs do not produce the desired effect in the expected time which branded ones do. That is why doctors are reluctant to use them. Regulatory authorities need to provide more emphasis on quality control of generic medicines.” (P4).*

## Discussion

Universal access to medicines, a critical component of Sustainable Development Goals, is intended to ‘ensure access to safe, effective, quality and affordable essential medicines for all [24]. Unfortunately, access to medicine is mainly determined by socioeconomic status in low and middle income countries, with poor medicine access among disadvantaged populations []. Indians mainly rely on private purchases for healthcare including medicine, and the country’s low public spending on health at 1.3% of GDP coupled with majority of people being either uninsured or underinsured makes this especially unaffordable for the poor [25]. Purchase of medicine contributes the most (60.6%) to the OOP health payments by households, and pushes more than 3% Indians into poverty every year [3, 25]. In response to poor medicine access and reduce the financial burden of medicine, Government of India has been promoting the use of unbranded generics by rebranding and expanding its generic medicine scheme (PMBJP).

We found that the overall availability of surveyed essential medicines was 47% in PMBJP outlets. Although the extent of availability of medicines seems to have improved at PMBJP stores, studies reported better availability in private sector in India and other LMICs [23, 26]. The following reasons might explain the lack of generic medicines in PMBJP stores. The discussion with the key stakeholders including store owners and pharmacists at the PMBJP stores revealed the gaps in supply chain management. It may be noted that BPSU procures and distributes medicines to all PMBJP outlets through a central warehouse located in Delhi. Issues such as lack of monitoring at warehouses, fewer regional drug warehouses for ensuring timely supplies to PMBJP stores, inadequate number of medicine suppliers, poor coordination between suppliers and PMBJP outlet owners, irregular training of PMBJP pharmacists to use PMBJP online portal effectively for updating stock situation on a real-time basis for maintaining an adequate supply and supply of generic medicines with short term expiry period have been cited as some of the reasons for low availability of common medicines at the PMBJP. Aside from the supply chain issues, relatively lower mark-up (20%) for selling PMBJP medicines as compared to the profit margins of branded-generic medicines and a meagre compensation against expiry of medicines (2% of total sales or actual loss) have been attributed to the high stock-outs of some common essential medicines [27],

One of the key findings of our study is that PMBJP list included only 214 essential drugs, implying that it excluded more than 50% of drugs listed under NLEM, 2015. Furthermore, FDC drugs are included in various therapeutic categories in PMBJP list. The inclusion of FDCs of antimicrobials, FDCs for CVDs and other diseases pose a public health concern. It is worth noting that though considerable research showed issues with some antibiotic FDCs, these FDCs still find their way to the Indian market [28]. Almost 70% of antibiotic FDCs available on the market are not registered with Central Drugs Standard Control Organisation (CDSO) [29]. In view of growing antibiotic resistance and concerns regarding safety and efficacy of FDCs manufactured in India, it is worrying to find that the use of FDCs is being promoted by PMBJP. The FDCs, except the ones of proven quality, should be removed from the PMBJP’s list of medicines to safeguard public health.

We found that switching from branded-generic medicine to unbranded generic medicines would lead to substantial cost savings for medicine consumers. Our estimates suggest that unbranded generic substitution would reduce pharmaceutical expenditure by 6–1129% of patient’s spending. Moreover, the unbranded generic medicines are found to be fairly affordable. These results are consistent with evidence from studies in other LMICs [30–32]. However, caution must be exercised while interpreting affordability because of the following reasons. First, affordability might differ depending upon the compliance of the minimum wages act of the Maharashtra government. Second, the level of economic development varies considerably across states and between rural and urban areas in India and this needs to be taken into account while extrapolating results of this study to other states and national level. Third, the unemployment rate is quite high (6.5%) and absolute poverty is widespread in the country (23%) [33]. As a large section of the population continues to live in hand to mouth condition, the PMBJP medicines may still be unaffordable to many people.

Making medicine accessible at affordable prices to the masses will require more than the somewhat piecemeal approach to generic drug promotion that currently exists in the form of PMBJP scheme. The sale of PMBJP medicines is mainly dependent on the prescription of generic drugs by the physicians. But doctors from private health care facilities hardly mention the generic or chemical names of drugs on the prescription slips and only 60 percent of the prescriptions from public health facilities contain generic names [27]. We found that, in spite of the awareness campaign by the PMBJP, physicians, especially those having private practice remained apprehensive regarding the therapeutic efficacy and the quality of unbranded generic medicines. Similar findings were also reported by studies in LMICs [34–36]. Besides quality concerns, generic medicine prescription seems to be also affected by other factors including the existence of a wide variety of alternative options of medicines available for a given disease, physicians’ fear of losing drug selection power to pharmacists due to mandatory generic prescribing, and lack of information about availability of generic medicines in PMBJP pharmacies among healthcare professionals, etc.

Although India’s case is lot more complex than most countries given the dominating presence of ‘branded’ generics in the Indian pharmaceutical market, it is worth learning from countries which achieved reasonable success in promoting the utilisation of generics. USA, the first country to implement a generic drug implementation policy, achieved 89% share of generic drugs in 2016 and this reduced the medical insurance expenditure by US$ 67.7 billion [37]. Japan, Canada, Australia, European countries as well as LMICs such as Philippines had similar experience regarding generic drug use [38, 39]. All these countries have either made it compulsory to prescribe drugs with active substance names or passed laws for mandatory generic substitution. Also, a formulary containing the details of therapeutically interchangeable products is available for reference [36]. And for ensuring the quality of generics, information regarding bioequiavailability is sought from companies before they place the products on the market. This practice has also been adopted by countries like China, and Malaysia [40, 41]. Aside from the supply side initiatives, demand side measures such as physicians’ motivation to prescribe generic drugs, consumers’ attitude towards generic drugs have shown some results [42–44].

### Limitations of this study

Although we reported availability, stock-outs and affordability of medicines in the PMBJP pharmacy outlets, a more comprehensive assessment of the accessibility of essential medicines may have included measurement of medicine prices, prescription pattern and out-of-pocket payments on medicines. In fact, we did carry out prescription audit as part of the overall study, but its findings are not included here. The present study adopted descriptive cross sectional research design to assess the current situation of PMBJP scheme in two districts of Maharashtra. However, to fully understand whether rebranding of JAS as PMBJP improved the accessibility of essential medicines, a pre/post evaluation study would have provided better insights but that could not be performed due to the non-availability of comparable baseline survey data.

Further, we did not fully follow WHO-HAI methodology for calculating availability of essential medicines as the list of surveyed medicines was modified to meet the contextual requirements. Besides, we did not compare the prices of unbranded generics with international reference prices as recommended by WHO-HAI methodology since there is already overwhelming evidence showing that generic prices in India are less expensive compared with international standards. Lastly, this study was restricted to two districts of Maharashtra state. Thus, the scope for generalisability of the study findings, especially on availability and affordability of PMBJP medicines is limited.

## Conclusion

The low availability of medicines at PMBJP outlets reflects the implementation issues faced by the scheme. Nevertheless, the study findings show that PMBJP’s unbranded generics offer great opportunities for substantial cost savings. But, in order to fully realise the potential of this scheme, some policy actions are urgently required. First, the PMBJP drug list must include all essential drugs that feature in NLEM. Second, BPPI should procure only those drugs that pass the bioequivalence test. Third, compulsory de-branding of generics should be done in a phased manner. Fourth, PMBJP’s medicine procurement and distribution policies must be reviewed to address the supply chain issues.

Moreover, there is a need for major pharmaceutical policy reforms to promote generic medicines in a big way. Regulations to support mandatory generic prescribing and generic substitution by pharmacists are needed. Besides, CDSO must ensure that generic medicines available in the Indian pharmaceutical market are bioequivalent and those qualifying bioequivalence tests should be listed along with their branded counterparts and price on a public or PMBJP portal.

## Supplementary Information

**Additional file 1. **Data collection tool – Availability and stock out duration of medicines.

**Additional file 2: Table A2.** Profile of Medical practitioners involved in study.

**Additional file 3: Table A3.** Details of Pharmacists.

## Data Availability

The data used and/or analysed during the current study are not publicly available. The datasets, including the qualitative dataset concern the implementation of a government scheme related with specific places and actors, making anonymising and removing any potentially sensitive observations, especially from interview transcripts difficult. However, they can be obtained from the corresponding author on reasonable request.

## Abbreviations

PMBJP: Pradhan Mantri Bhartiya Jan Aushadhi Pariyojna
UPA: United Progressive Alliance
NLEM: National list of Essential Medicines
BPPI: Bureau of Public Pharmaceutical Undertakings
FDCs: Fixed Dose Combinations
UHC: Universal Health Coverage
WHO-HAI: World Health Organization- Health Action International
PHC: Primary Health Centre
HP: Health Post
CVD: Cardiovascular Diseases
NCDs: Non Communicable Diseases
DPCO: Drug (Price Control) Order
NPPA: National Pharmaceutical Pricing Authority
MBP: Market Based Pricing
LMICs: Low and Middle-Income Countries

## Footnotes

1 The DPCO is an order issued by the government under the essential commodities act which enables it to fix the prices of some essential bulk drugs and their formulations.

2 Exchange rate: $$1 USD=74.97 rupees$$

3 The price cap is calculated by taking the simple average of prices of all brands and generic versions of the medicine having market share more than or equal to 1% of the total market turnover of that medicine.

4 In India, branded generic medicine has the largest market share (90%), followed by trade generics (8%), and unbranded generics as well as original brand-name drugs (both 1% share) [Srinivas 2014]. The existence of these three types of generics is basically the result of marketing strategies employed by the pharma companies to maximise their revenue earnings. Although unbranded generic medicine is the cheapest of all, its penetration in the retail pharmaceutical market is limited to PMBJP stores. The demand for unbranded generics is mainly driven by the government procurement for meeting the requirement of the public health system.
