# Supplementary material for: Improving access to medicines by popularising generics: a study of ‘India’s People’s Medicine’ scheme in two districts of Maharashtra
Source: BMC Health Serv Res. 2022 May 13;22:643. doi: 10.1186/s12913-022-08022-1 (PMC9107149; doi:10.1186/s12913-022-08022-1)
Supplement: Supplementary file 1 — Additional file 1. Data collection tool – Availability and stock out duration of medicines. [file 12913_2022_8022_MOESM1_ESM.docx]

|  | **Data collection tool – Availability and stock out duration of medicines** | | | | | | |
| --- | --- | --- | --- | --- | --- | --- | --- |
| **Sr.No** | **Therapeutic Category** | **Drug Name** | **Level of Healthcare** | **Type of formulation** | **Strength** |  |  |
|  |  |  |  |  |  | **Availability (Yes/No)** | **No of days of stock outs** |
|  | **Antimicrobial** | Acyclovir | P, S, T | Tab | 200 mg |  |  |
|  |  | Amoxicillin | P, S, T | Cap | 250 mg |  |  |
|  |  | Amoxicillin (A) + Clavulanic acid (B) | P,S,T | Tab | 500 mg (A) + 125 mg (B) Oral |  |  |
|  |  | Ceftriaxone | S, T | Inj | 1 g powder |  |  |
|  |  | Ciprofloxacin | P, S, T | Tab | 500 mg |  |  |
|  |  | Co-trimoxazole [Sulphamethoxazol e (A) +  Trimethoprim (B)] | P, S, T | Suspension | Oral liquid 200 mg (A) + 40 mg (B)/5 ml |  |  |
|  |  | Fluconazole | S, T | Inj | Injection 200 mg /100 ml |  |  |
|  | **Cardiovascular Medicine** | Atenolol | P, S, T | Tab | 50 mg |  |  |
|  |  | Enalapril | P, S, T | Tab | 5 mg |  |  |
|  |  | Clopidogrel | T | Tab | 75 mg |  |  |
|  |  | Atorvastatin | P,S,T | Tab | 10 mg |  |  |
|  |  | Telmisartan | P, S, T | Tab | 20 mg |  |  |
|  |  | Nifedipine | S, T | Tab | 10 mg |  |  |
|  | **Antidiabetics** | Glimepiride | P, S, T | Tab | 1 mg |  |  |
|  |  | Metformin | P, S, T | Tab | 500 mg |  |  |
|  |  | Premix Insulin 30:70 Injection (Regular:NPH) | P, S, T | Injection | 40 IU/ml |  |  |
|  | **Medicines used in Palliative care** | Amitriptyline | T | Tab | 25 mg |  |  |
|  |  | Diazepam | T | Tab | 5mg |  |  |
|  | **Antineoplastic/ Anticancer** | Cyclophosphamide | T | Tab | 50 mg |  |  |
|  |  | Cisplatin | T | Injection | 1mg/ml |  |  |
|  |  | Imatinib | T | Tab | 100 mg |  |  |
|  | **Psychological Disorder** | Fluoxetine | T | Cap | 20 mg |  |  |
|  |  | Fluphenazine | S, T | Depot Inj | 25mg/ml |  |  |
|  | **Antiepileptics** | Carbamazepine | P, S, T | Tab | 200 mg |  |  |
|  |  | Phenytoin | P, S, T | Tab | 100 mg |  |  |
|  | **Antiasthamatic** | Budesonide | P, S, T | Inhaler | Inhalation (MDI/DPI) 100 mcg/dose |  |  |
|  |  | Salbutamol | P, S, T | Inhaler | Oral liquid 2 mg/5 ml Inhalation (MDI/DPI) 100 mcg/dose |  |  |
|  | **Analgesic** | Diclofenac | P,S,T | Tab | 50 mg |  |  |
|  |  | Tramadol | S,T | Cap | 50 mg |  |  |
|  | **Antacids** | Omeprazole | P, S, T | Cap | 20 mg |  |  |
|  |  | Ranitidine | P, S, T | Tab | 150 mg |  |  |
|  | **Medicines for electrolyte balance** | Oral rehydration Salt | P, S, T | Powder | As licensed |  |  |
|  |  | Sodium chloride | S, T | Injection | 0.45% / 3% |  |  |
|  | **Vitamins** | Ascorbic acid (Vitamin C) | P, S, T | Tab | 100 mg |  |  |
|  |  | Calcium carbonate | P, S, T | Tab | 250 mg |  |  |
|  | **Consumables** | Glucometer test strip |  |  |  |  |  |
|  |  | Glucometer digital(1 glucometer- 25 strips, 15 lancets,1 lancing device,1battery 3v. |  |  |  |  |  |

Name of PMBJP Kendra:

Date of Visit:
