# Supplementary material for: Improving access to medicines by popularising generics: a study of ‘India’s People’s Medicine’ scheme in two districts of Maharashtra
Source: BMC Health Serv Res. 2022 May 13;22:643. doi: 10.1186/s12913-022-08022-1 (PMC9107149; doi:10.1186/s12913-022-08022-1)
Supplement: Supplementary file 2 — Additional file 2: Table A2. Profile of Medical practitioners involved in study. [file 12913_2022_8022_MOESM2_ESM.docx]

**Table.A2 Profile of Medical practitioners involved in study**

| **Participant’s ID** | **Sex** | **Qualification** | **Sector** |
| --- | --- | --- | --- |
| PuP1 | Male | Postgraduate/ MD | Public Hospital |
| PuP2 | Female | Postgraduate/ MD | Public Hospital |
| PuP3 | Male | Postgraduate/ MD | Public Hospital |
| PrP1 | Male | Graduate/ MBBS | Private Clinic |
| PrP2 | Male | Graduate /MBBS | Private Clinic |
| PrP3 | Female | Graduate/ MBBS | Private Clinic |

PuP: Public Physician, PrP: Private Physician
