# Supplementary material for: Improving access to medicines by popularising generics: a study of ‘India’s People’s Medicine’ scheme in two districts of Maharashtra
Source: BMC Health Serv Res. 2022 May 13;22:643. doi: 10.1186/s12913-022-08022-1 (PMC9107149; doi:10.1186/s12913-022-08022-1)
Supplement: Supplementary file 3 — Additional file 3: Table A3. Details of Pharmacists. [file 12913_2022_8022_MOESM3_ESM.docx]

**Table A3. Details of Pharmacists**

| **Participant’s ID** | **Sex** | **Qualification** |
| --- | --- | --- |
| P1 | Male | Bachelor in Pharmacy |
| P2 | Male | Bachelor in Pharmacy |
| P3 | Male | Bachelor in Pharmacy |
| P4 | Male | Diploma in Pharmacy |
| P5 | Male | Bachelor in Pharmacy |
| P6 | Male | Diploma in Pharmacy |
| P7 | Male | Diploma in Pharmacy |
| P8 | Male | Bachelor in Pharmacy |
| P9 | Male | Bachelor in Pharmacy |
| P10 | Male | Bachelor in Pharmacy |
